# Pharmacological treatment of patients with chronic heart failure. Subanalysis of an Ecuadorian registry

**DOI:** 10.47487/apcyccv.v6i2.470

**Published:** 2025-06-27

**Authors:** Luis Moreno-Rondón, María Elizabeth Ortega-Armas, Diego Pulla, Robert Alarcón Cedeño, Juan Díaz Heredia, Diego Villavicencio, Oscar Luces-Tejada, Mario Gómez, Alex Castro-Mejía

**Affiliations:** 1 Hospital General del Norte de Guayaquil «Los Ceibos», Guayaquil-Ecuador. Hospital General del Norte de Guayaquil «Los Ceibos» Guayaquil Ecuador

**Keywords:** Heart Failure, Treatment, Prognosis, Insuficiencia Cardiaca, Tratamiento, Pronóstico

## Abstract

**Introduction.:**

Introduction. In Ecuador, there is limited data on the treatment of patients with heart failure (HF).

**Objective.:**

This study aimed to determine the rate of use of prognosis-modifying drugs and their association with prognosis.

**Materials and methods.:**

A retrospective observational study was conducted on patients with chronic HF included in the “Los Ceibos” registry between January 2017 and December 2022. Patients were followed for a median of 2.28 years (interquartile range [IQR]: 1.25-3.49).

**Results.:**

A total of 711 patients diagnosed with HF were included. Among them, 82.7% (n=588) received angiotensin-converting enzyme inhibitors (ACEIs), angiotensin receptor blockers (ARBs), or angiotensin receptor-neprilysin inhibitors (ARNIs); 82.3% (n=585) received beta-blockers (BBs); and 51.3% (n=365) were treated with mineralocorticoid receptor antagonists (MRAs). Among patients with HFrEF, those receiving triple therapy (ACEI/ARB/ARNI + BB + MRA) had lower all-cause mortality compared to other groups (38.8%, log-rank p=0.014). In patients with Heart Failure with preserved Ejection Fraction (HFpEF), no mortality differences were observed according to the number of medications used (log-rank p=0.720). MRA use was not associated with a prognostic benefit in HFpEF (p>0.05). Patients receiving triple therapy with ARNI + BB + MRA had better survival during follow-up compared to any other drug combination (log-rank p=0.027).

**Conclusions.:**

A high rate of ACEI/ARB/ARNI and BB use was observed. The use of triple therapy, particularly the combination of ARNI + BB + MRA, was associated with improved prognosis in patients with HFrEF over a four-year follow-up period. No prognostic benefit of MRA use was observed in patients with HFpEF.

## RESUMEN

**Introducción.:**

En Ecuador existen pocos datos acerca del tratamiento de los pacientes con insuficiencia cardiaca (IC).

**Objetivo.:**

Este estudio tiene como objetivo determinar la tasa de uso de medicamentos modificadores del pronóstico y su relación con el pronóstico.

**Materiales y métodos.:**

Estudio observacional retrospectivo en pacientes con IC crónica, del registro «Los Ceibos» durante el periodo enero de 2017 a diciembre de 2022 y se realizó seguimiento por un tiempo medio de 2,28 (RIQ 1,25-3,49) años**.**

**Resultados.:**

Se incluyeron 711 pacientes con el diagnóstico de IC. El 82,7 % (n=588) recibió inhibidores de la enzima convertidora de angiotensina (IECA), antagonistas de los receptores de angiotensina II (ARA) o inhibidores duales de la neprilisina y del receptor de angiotensina (INRA). El 82,3% (n=585) recibió betabloqueantes (BB) y el 51,3% (n=365) recibió antagonistas de los receptores de mineralocorticoide (ARM). Los pacientes que tuvieron triple terapia (IECA/ARA/INRA+BB+ARM) tuvieron menor mortalidad por todas las causas en IC-FEr (38,8%, p Log Rank=0,014); en IC-FElr no se observó diferencias en la mortalidad en cuanto al número de fármacos (p Log Rank=0,720). El uso de ARM no se relacionó con un beneficio pronóstico en IC-FEp (p>0,05). Los pacientes que recibieron triple terapia con INRA+BB+ARM tuvieron mejor supervivencia en el seguimiento comparado con cualquier otra combinación de fármacos en IC-FEr (p Log Rank=0,027).

**Conclusiones.:**

Se observó una alta tasa de uso de IECA/ARA/INRA y BB. El empleo de tres fármacos de la terapia fundacional, específicamente la combinación de INRA+BB+ARM, se relacionó con mejor pronóstico en pacientes con IC-FEr a cuatro años de seguimiento. No se observó un beneficio pronóstico de ARM en IC-FEp.

## Introduction

Heart failure (HF) is a condition associated with high morbidity and mortality both nationally and globally ^1^^,^^2^. Worldwide, more than 60 million people live with HF. Its prevalence is closely linked to age, affecting less than 2% of people under 60 years of age and more than 10% of those over 75. This age-dependent increase in prevalence is likely due to population ageing and improved effectiveness of current medical therapies. Annual healthcare expenditures for HF exceed USD 100 billion, with approximately half of this amount attributed to hospitalised patients ^2^^-^^4^.

In real-world practice, the prescription and titration of disease-modifying therapies for HF often fall far short of guideline-recommended targets ^5^. For example, the "Change the Management of Patients with Heart Failure" (CHAMP-HF registry) found that among eligible patients, only 1% were receiving a combination of guideline-directed medical therapies at optimal doses ^6^. In a more recent registry involving a database of 17,000 patients, it was reported that one year after hospital discharge for HF, only 13% of patients were receiving triple therapy, while up to 23% were not receiving any foundational therapy at all ^7^.

Most of the available data on HF treatment come from large-scale studies with limited representation from low- and middle-income countries ^8^. In Ecuador, there is scarce information regarding the pharmacological management of patients with HF. The primary objective of this study is to describe the use of medications with proven prognostic benefit, as recommended by current clinical practice guidelines for HF ^9^. The secondary objective is to analyse differences in clinical outcomes during follow-up according to the type of treatment administered across different strata of left ventricular ejection fraction (LVEF).

## Materials and methods

### Study design

“Los Ceibos” HF registry is a retrospective, single-centre observational study that includes outpatients with HF seen at the outpatient clinic of the Hospital General del Norte de Guayaquil “Los Ceibos” between January 2017 and December 2022. The main design features and primary results of the registry have been published previously ^1^. 

### Study population

All patients aged ≥ 18 years with symptoms and signs of HF, along with structural abnormalities on echocardiography and/or elevated N-terminal pro-B-type natriuretic peptide (NT-proBNP) levels ^10^^-^^12^, were included if they had a clinical diagnosis of HF as determined by the treating physician, regardless of LVEF. Patients were identified using the following International Classification of Diseases, 10th Revision (ICD-10) codes: I50 (congestive HF), I50.9 (HF, unspecified), I42.0 (dilated cardiomyopathy), and I11 (hypertensive heart disease with HF). Patients with severe primary valvular disease and those with restrictive or hypertrophic cardiomyopathies were excluded to avoid diagnostic confounding, particularly in cases with preserved ejection fraction, as described by Kittleson *et al.*^11^. Data collection was conducted prior to the approval of sodium-glucose cotransporter 2 inhibitors (SGLT2 inhibitors) for the treatment of HF in Ecuador.

### Variables

Demographic variables and comorbidities analysed included age, sex, hypertension, diabetes mellitus, dyslipidaemia, obesity, ischaemic heart disease, atrial fibrillation, chronic kidney disease, dialysis, cerebrovascular disease, and chronic obstructive pulmonary disease. Clinical variables included angina, dyspnoea, palpitations, peripheral oedema, and functional class according to the New York Heart Association (NYHA) classification.

Treatment-related variables included the use or non-use of: angiotensin-converting enzyme inhibitors (ACEis), angiotensin receptor blockers (ARBs), angiotensin receptor-neprilysin inhibitors (ARNIs), beta-blockers (BBs), mineralocorticoid receptor antagonists (MRAs), digoxin, loop diuretics, amiodarone, nitrates, thiazides, calcium channel blockers, statins, antiplatelet agents, anticoagulants, coronary revascularisation, and cardiac device therapy.

Laboratory parameters included haemoglobin, creatinine, estimated glomerular filtration rate, and NT-proBNP (all measured at the first medical visit upon inclusion in the study). Echocardiographic variables included left ventricular end-diastolic diameter, LVEF, moderate-to-severe mitral regurgitation, moderate-to-severe tricuspid regurgitation, and pulmonary hypertension.

Clinical outcomes of interest were hospital admission for any cause, admission for HF, all-cause mortality, and cardiovascular mortality. Cardiovascular mortality was defined as death due to acute coronary syndrome, HF, cardiogenic shock, arrhythmias, or sudden cardiac death.

### Procedures or interventions

Data were collected from electronic medical records. Patients with insufficient information regarding comorbidities, treatment, or prognosis were excluded from the analysis.

HF with preserved ejection fraction (HFpEF) was defined as LVEF ≥ 50%; HF with mildly reduced ejection fraction (HFmrEF) as LVEF between 41-49%; and HF with reduced ejection fraction (HFrEF) as LVEF ≤ 40%.

For the prognostic analysis of treatment, the following were defined as disease-modifying therapies: 1) renin-angiotensin-aldosterone system inhibitors (RAASi), including ACEis, ARBs, or ARNIs; 2) BBs; and 3) MRAs. SGLT2i were not included in the analysis, as this study was conducted prior to their approval in Ecuador (13). In patients with HFpEF, only the use of MRAs was evaluated.

### Ethical aspects

This study adheres to the principles of the Declaration of Helsinki and was approved by an ethics committee for research involving human subjects. Data were obtained from the AS400 (IBM) electronic medical record system of the Ecuadorian Social Security Institute. Informed consent was not required, as data were collected from a secondary source. All individuals in the database underwent an anonymisation process to ensure privacy.

### Data analysis

Continuous variables were expressed as mean ± standard deviation (SD) or median with interquartile range (IQR), and categorical variables were presented as frequencies and percentages. Comparisons of categorical variables were made using the chi-squared test, while comparisons of continuous variables were conducted using ANOVA.

Clinical event analyses were performed using the Kaplan-Meier method, with group comparisons assessed using the log-rank test.

All p-values were two-tailed, with 95% confidence intervals (95% CI), and values of p<0.05 were considered statistically significant. Statistical analyses were performed using STATA version 18 (Stata Corp., College Station, TX, USA).

## Results

Between January 2017 and December 2022, a total of 711 patients with a diagnosis of HF were included. Of these, 333 (46.8%) had HFrEF, 109 (15.3%) had HFmrEF, and 269 (37.8%) had HFpEF. Overall, 82.7% (n = 588) received ACEis, ARBs, or ARNIs; 82.3% (n = 585) received BBs; 51.3% (n = 365) received MRAs; and 51.1% (n = 363) received loop diuretics.

Among patients receiving ACEis, ARBs, or ARNIs, 70.6% were aged ≥ 65 years; most had hypertension (92.7%), 40.1% had diabetes, 24.5% had ischaemic heart disease (IHD), and 30.6% had chronic kidney disease (CKD). Similar proportions were observed in the BBs and MRAs groups (Table 1).

**Table 1:** Clinical characteristics and treatment of patients in “Los Ceibos” heart failure registry

	Total n=711	ACEi/ARB/ARNI n=588 (82,7%)*	BB n=585 (82,3%)*	MRA n=365 (51,3%)*	Loop diuretic n=363 (51,1%)*
Comorbidities					
Age, mean ± SD	69.8±13.1	70.7±12.5	69.7±13.3	69.3±13.6	71.2±13.0
≥65 years	491 (69.1)	415 (70.6)	406 (68.4)	253 (69.3)	268(73.8)
Women, n (%)	223 (31.4)	180 (30.6)	396 (67.7)	250 (68.5)	243 (66.9)
Hypertension, n (%)	658 (92.7)	555 (94.4)	544 (93.0)	336 (92.1)	343 (94.5)
Diabetes *mellitus*, n (%)	275 (38.7)	236 (40.1)	233 (39.8)	141 (38.6)	149 (41.0)
Dyslipidaemia, n (%)	318 (44.7)	270 (45.9)	269 (46.0)	173 (47.4)	174 (47.9)
Obesity	200 (29.3)	179 (30.4)	173 (29.6)	108 (29.6)	111 (30.6)
Ischaemic heart disease, n (%)	174 (32.2)	144 (24.5)	146 (25.0)	93 (25.5)	80 (22.0)
Atrial fibrillation, n (%)	180 (25.3)	152 (25.9)	154 (26.3)	107 (29.3)	107 (29.5)
Chronic kidney disease, n (%)	211 (29.7)	180 (30.6)	179 (30.6)	108 (29.6)	136 (37.5)
Dialysis, n (%)	67 (9.4)	54 (9.2)	58 (9.9)	22 (6.0)	38 (10.5)
Cerebrovascular disease, n (%)	81 (11.4)	76 (12.9)	67 (11.5)	44 (12.1)	44 (12.1)
COPD, n (%)	31 (4.4)	27 (4.6)	20 (3.4)	19 (5.2)	19 (5.2)
Clinical presentation, n (%)					
Angina	170 (23.9)	136 (23.2)	145 (24.8)	85 (23.4)	94 (26.0)
Dyspnoea	430 (60.6)	368 (62.7)	368 (63)	254 (69.8)	263 (72.7)
Palpitations	106 (20.0)	115 (19.6)	115 (19.7)	81 (22.3)	84 (23.2)
Peripheral oedema	207 (29.2)	178 (30.4)	177 (30.4)	128 (35.3)	153 (42.3)
NYHA Class I-II	572 (80.5)	456 (77.6)	442 (75.6)	260 (71.2)	255 (70.2)
NYHA Class III-IV	139 (19.5)	132 (22.4)	143 (24.4)	105 (28.8)	108 (29.8)
Other treatments					
ACEi/ARB/ARNI , n (%)	588 (82.7)	-	491 (83.9)	323 (88.5)	306 (84.3)
ARNI, n (%)	50 (7.0)	-	46 (7.9)	35 (9.6)	33 (9.1)
BB, n (%)	585 (82.3)	491 (83.5)	-	331 (90.7)	320 (88.2)
MRAs, n (%)	365 (51.3)	323 (54.9)	331 (56.6)	-	229 (63.1)
Digoxin, n (%)	62 (8.7)	51 (8.7)	55 (9.4)	47 (12.9)	47 (12.9)
Loop diuretics, n (%)	363 (51.1)	306 (52.0)	320 (54.7)	229 (62.7)	-
Amiodarone, n (%)	59 (8.3)	48 (8.2)	44 (7.5)	36 (9.9)	33 (9.1)
Nitrates, n (%)	25 (3.5)	22 (3.7)	21 (3.6)	10 (2.7)	11 (3.0)
Thiazides, n (%)	109 (15.3)	102 (17.2)	91 (15.6)	65 (17.8)	44 (12.1)
Calcium channel blockers, n (%)	196 (27.6)	178 (30.3)	164 (28.0)	92 (25.2)	107 (29.5)
Statins, n (%)	202 (28.4)	180 (30.6)	174 (29.7)	115 (31.5)	122 (33.6)
Antiplatelets, n (%)	395 (55.6)	337 (57.3)	345 (59.0)	204 (55.9)	198 (54.5)
Anticoagulants, n (%)	219 (30.8)	190 (32.3)	192 (32.8)	142 (38.9)	142 (39.1)
Revascularisation (PCI/CABG), n (%)	145 (20.4)	120 (20.4)	123 (21.0)	69 (18.9)	58 (16.0)
Devices (PM/ICD/CRT), n (%)	87 (12.2)	67 (11.4)	72 (12.3)	52 (14.2)	50 (13.8)

*Percentage of the total study population. SD: standard deviation. ACEi: angiotensin-converting enzyme inhibitor. ARB: angiotensin receptor blocker. ARNI: angiotensin receptor-neprilysin inhibitor. BB: beta-blocker. MRA: mineralocorticoid receptor antagonist. PCI: percutaneous coronary intervention. CABG: coronary artery bypass grafting. PM: pacemaker. ICD: implantable cardioverter defibrillator. CRT: cardiac resynchronisation therapy. COPD: chronic obstructive pulmonary disease.

**Figure 1:**
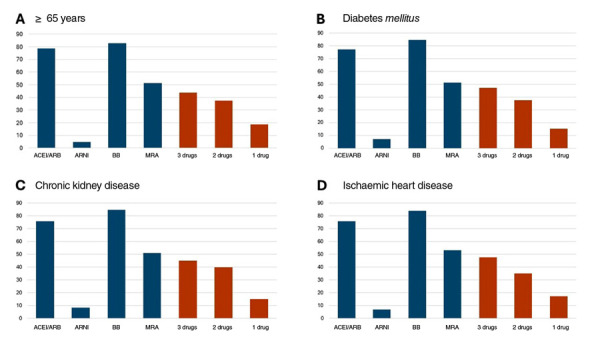
Prescription rates of medications across major comorbidities. Blue bars indicate the use of each drug, whether alone or in combination. Orange bars represent the use of specific combinations of prognostic-modifying therapies.

The use rate of ACEis/ARBs was approximately 80% among patients aged ≥ 65 years and those with diabetes, CKD, or IHD. Among patients aged ≥ 65 years, the use of ARNIs was limited to 4.9%, while in those with CKD, this rate was 8.5%. The most common treatment combination was triple therapy with an ACEis/ARB/ARNI + BB + MRA, prescribed in 47.3% of patients with diabetes, 45% of those with CKD, and 47.7% of patients with IHD (Figure 1).

Patients using loop diuretics had a lower estimated glomerular filtration rate (56.6 ± 29.4 mL/min/1.73 m²). Among MRA users, the proportion of patients with HFrEF was higher (62.7%, n = 229), while those with HFpEF were fewer (24.4%, n = 89), compared with users of ACEis/ARBs/ARNIs or BBs (Table 2).

**Table 2:** Complementary tests.

	Total n=711	ACEi/ARB/ARNI n=588 (82,7%)*	BB n=585 (82,3%)*	MRAs n=365 (51,3%)*	Loop diuretics N=363 (51,1%)*
Laboratory (mean ± SD)				
Haemoglobin (g/dL)	12.6±2.3	12.6±2.5	12.6±2.3	12.6±2.2	12.4±2.3
Creatinine (mg/dL)	1.7±1.6	1.6±1.6	1.7±1.7	1.6±1.4	1.8±1.7
eGFR (mL/min/1,73 m2)	61.2±29.3	64.8±30.1	60.5±29.0	61.7±28.5	56.6±29.4
NT-ProBNP (pg/mL) (n=369)	6655.7±1179.7	3620.0±748	6564.6±975	9967.7±715	7951.6±1486
Echocardiography					
LVEDD (mm), mean ± SD	54.3±11.1	48.2±6.8	51.6±8.9	56.9±12.4	55.3±11.1
LVEF (%), mean ± SD	43.3±15	59.7±6.2	42.1±14.7	38.1±14.7	39.9±14.9
HFrEF, n (%)	333 (46.8)	285 (48.5)	291 (49.7)	229 (62.7)	205 (56.5)
HFmrEF, n (%)	109 (15.3)	89 (15.1)	89 (15.2)	47 (12.9)	54 (14.9)
HFpEF, n (%)	269 (37.8)	214 (36.4)	205 (35.0)	89 (24.4)	104 (28.7)
Moderate/severe MR, n (%)	226 (31.8)	195 (33.2)	180 (30.8)	116 (31.8)	116 (32.0)
Moderate/severe TR, n (%)	157 (22.1)	127 (21.6)	134 (22.9)	91 (24.9)	101 (27.8)
Pulmonary hypertension, n (%)	252 (37.2)	209 (35.5)	219 (37.4)	152 (41.6)	149 (41.0)

*Percentage of the total study population. ACEi: angiotensin-converting enzyme inhibitor. ARB: angiotensin receptor blocker. ARNI: angiotensin receptor-neprilysin inhibitor. BB: beta-blocker. MRA: mineralocorticoid receptor antagonist. eGFR: estimated glomerular filtration rate. NT-ProBNP: N-terminal pro-B-type Natriuretic Peptide. HFrEF: heart failure with reduced ejection fraction. HFmrEF: heart failure with mildly reduced ejection fraction. HFpEF: heart failure with preserved ejection fraction. LVEDD: left ventricular end-diastolic diameter. LVEF: left ventricular ejection fraction. MR: mitral regurgitation. TR: tricuspid regurgitation.

In the general population, use of disease-modifying medications (ACEis/ARBs/ARNIs and/or BBs and/or MRAs) was distributed as follows: 18.7% received only one drug (133 patients), 36.4% received two drugs (259 patients), and 44.9% received all three (319 patients). Patient characteristics by number of drugs used are presented in Table 3.

**Table 3:** Clinical characteristics and events by number of prognostic drugs (ACEi/ARB/ARNI and/or BB and/or MRA).

	1 drug n=133	2 drugs n=259	3 drugs n=319	p-value
Clinical characteristics				
≥65 years	93 (69.9)	163 (70.7)	491 (69.1)	0.681
Women, n (%)	42 (31.6)	79 (30.5)	102 (32.0)	0.929
Hypertension, n (%)	122 (91.7)	249 (96.1)	287 (90.0)	0.018
Diabetes mellitus, n (%)	42 (31.6)	103 (39.8)	130 (40.8)	0.171
Obesity	35 (26.3)	81 (31.3)	91 (28.5)	0.565
Ischaemic heart disease, n (%)	30 (22.6)	61 (23.6)	83 (26.0)	0.672
Atrial fibrillation, n (%)	27 (20.3)	69 (26.6)	84 (26.3)	0.336
Chronic kidney disease, n (%)	32 (24.1)	84 (32.4)	95 (29.8)	0.228
Clinical events				
HFrEF				
All-cause admission, n (%)	17 (51.5)	42 (44.2)	85 (41.3)	0.526
Admission for HF, n (%)	7 (21.1)	17 (17.9)	52 (25.2)	0.359
All-cause mortality, n (%)	20 (60.6)	51 (53.7)	80 (38.8)	0.010
Cardiovascular mortality, n (%)	10 (30.3)	21 (22.1)	55 (26.7)	0.573
HFmrEF				
All-cause admission, n (%)	6 (33.3)	19 (38.0)	12 (30.0)	0.726
Admission for HF, n (%)	2 (11.1)	3 (6.0)	5 (12.5)	0.547
All-cause mortality, n (%)	7 (38.9)	25 (50.0)	20 (50.0)	0.690
Cardiovascular mortality, n (%)	3 (16.7)	15 (30.0)	10 (25.0)	0.534
HFpEF*				
All-cause admission, n (%)	30 (33.7)	-	.	.
Admission for HF, n (%)	15 (16.9)	.	.	.
All-cause mortality, n (%)	25 (28.1)	.	.	.
Cardiovascular mortality, n (%)	12 (13.5)	.	.	.

*Only MRAs were included, as foundational therapy has not demonstrated efficacy in HFpEF.

HFrEF: heart failure with reduced ejection fraction. HFmrEF: heart failure with mildly reduced ejection fraction. HFpEF: heart failure with preserved ejection fraction.

**Figure 2:**
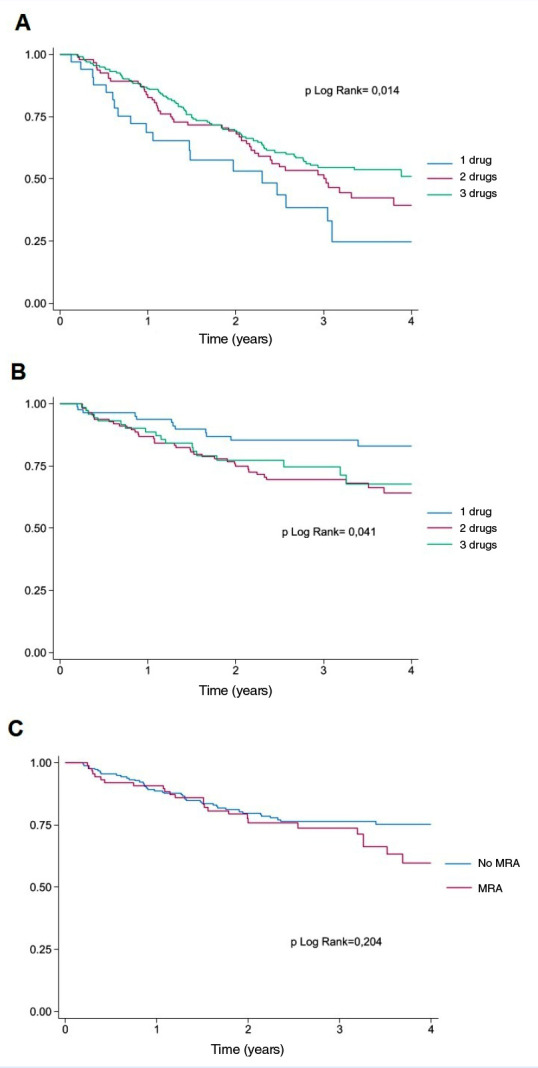
Kaplan-Meier survival curves according to the number of prognostic-modifying drugs. A. Reduced ejection fraction. B. Mildly reduced ejection fraction. C. Preserved ejection fraction.

During follow-up (median: 2.28 years, IQR: 1.25-3.49), 41.3% (n = 85) of HFrEF patients receiving triple therapy were hospitalised for any cause, a proportion not significantly different from those receiving one or two drugs (p = 0.526). However, all-cause mortality was lower in patients on triple therapy (38.8%) compared with those on two drugs (53.7%) or one drug (60.6%) (p = 0.010) (Table 3 and Figure 2A). In the HFmrEF group, no significant differences in event rates were observed during follow-up (p > 0.05). In the HFpEF group, only the use of MRAs was analysed; all-cause mortality was 28.1%, and cardiovascular mortality was 13.5%, with no significant differences compared to patients not receiving MRAs (p > 0.05) (Table 3 and Figure 2B).

**Figure 3:**
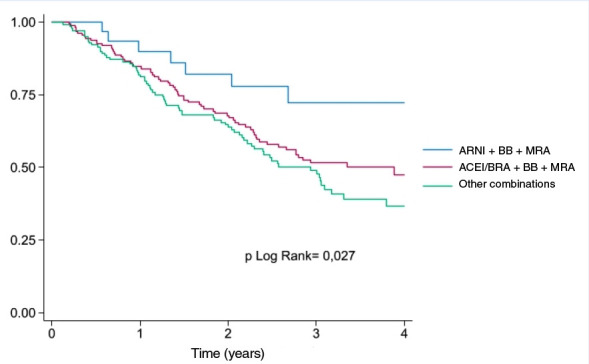
Survival curve in patients with HFrEF according to the type of pharmacological combination used.

Among patients with HFrEF, those treated with triple therapy consisting of ARNI + BB + MRA had better 4-year survival compared with those receiving ACEi/ARB + BB + MRA or other drug combinations (log-rank p = 0.027) (Figure 3).

## Discussion

In Ecuador, few registries provide insight into the pharmacological management of HF. The findings from the “Los Ceibos” chronic HF registry contribute to the understanding of treatment patterns in patients with HF, according to the current classification based on LVEF. The main findings were: 1) there is a high prescription rate of renin-angiotensin-aldosterone system inhibitors and BBs; 2) the use of all three foundational therapies was associated with improved prognosis in patients with HFrEF; 3) the combination of ARNI, BB, and MRA was associated with better outcomes compared to other combinations in patients with HFrEF; and 4) MRAs did not show prognostic benefit in patients with HFpEF.

The prescription rates of ACEis/ARBs/ARNIs and BBs in this registry exceeded 80% for both drug classes, similar to findings from another regional registry (84% for ACEis/ARBs/ARNIs and 79% for BB) ^14^, and comparable to those reported in a large European registry (86.5% for ACEis/ARBs/ARNIs and 89% for BB) ^15^.

Although ARNIs have been included in clinical practice guidelines since 2016 ^16^, their widespread adoption has not been achieved in Ecuador or globally. This is reflected in a study from Sweden, where ARNI use in patients with HFrEF increased from 8.3% in 2017 to 26.7% in 2021. Even in randomised clinical trials, ARNI use has remained suboptimal: in the “Empagliflozin in Patients with Chronic Heart Failure with Reduced Ejection Fraction” (EMPEROR-Reduced) trial, ARNI use was only 19.5% ^17^; in the “Dapagliflozin and Prevention of Adverse-Outcomes in Heart Failure” (DAPA-HF) trial, it was 10.7% ^18^; and in the “Vericiguat Global Study in Subjects with Heart Failure with Reduced Ejection Fraction” (VICTORIA) trial, only 14.5% of patients were receiving ARNIs at randomisation ^19^. In Ecuador, ARNIs are not included in the national essential medicines list, limiting their availability and uptake. According to data from this registry, ARNI use was 12% in patients with HFrEF, 9.2% in those with HFmrEF, and 0% in those with HFpEF ^1^. Moreover, some studies have questioned the benefit of ARNIs, particularly in patients with advanced disease and high functional class, as indicated by the LIFE trial ^20^.

Just over half of the patients were receiving MRAs. Although this proportion is far from optimal, it is comparable to that reported in other settings ^15^. The prescription rate may be influenced by the high proportion of patients with CKD included in our registry, as well as by physicians’ anticipation of potential complications associated with MRA use in this population.

Foundational therapy for HfrEF, which includes ARNIs, BB, MRAs, and SGLT2i, has been shown to significantly reduce mortality in clinical trials. According to a meta-analysis of 69 randomised clinical trials, the combination of these medications was associated with a reduction in all-cause mortality, with a hazard ratio (HR) of 0.36 (95% CI: 0.20-0.60) compared to placebo ^21^. Similar findings were observed in our registry, where all-cause mortality was lower among patients receiving the combination of ARNI + BB + MRA compared to any other drug combination. In our registry, half of the patients with HFrEF were receiving triple therapy. Due to the retrospective nature of this study, we were unable to determine the reasons for incomplete prescription in the remaining 50%; however, international registries have also reported suboptimal implementation of guideline-directed therapy, even in large-scale studies such as the “Evolution Heart Failure” registry ^22^. In patients with HFpEF, the use of MRAs was not associated with improved prognosis. Both ARNIs and MRAs have demonstrated some benefit in specific subgroups of patients with HFpEF, although the evidence regarding mortality reduction remains inconsistent ^23^^,^^24^. In the case of BB, there is no strong evidence supporting a reduction in hospitalisations among patients with HFpEF ^25^^-^^27^.

In our registry, ARNIs were not used in patients with HFpEF. This is even though sacubitril/valsartan has been evaluated for use in HFpEF in several recent studies and clinical guidelines. According to the 2023 ACC Expert Consensus Decision Pathway on Management of Heart Failure With Preserved Ejection Fraction, the use of ARNIs in HFpEF provides a modest additional benefit compared to valsartan alone, particularly in patients recently hospitalised and in specific subgroups, such as those with a LVEF between 45% and 57%, and in women ^28^. SGLT2i, which were not used in this registry, have been shown to improve outcomes in patients with HFpEF. Other drugs have also been investigated more recently in patients with HFmrEF and HFpEF. The “Finerenone Trial to Investigate Efficacy and Safety Superior to Placebo in Patients With Heart Failure” (FINEARTS-HF) assessed the efficacy and safety of finerenone, demonstrating that the drug significantly reduced the incidence of HF worsening and cardiovascular death compared to placebo ^29^. However, finerenone has not been evaluated in our registry.

The primary limitation of this study is its retrospective design and single-centre nature, which limit the applicability and generalisability of the findings. An additional limitation is the lack of data on SGLT2i use, as these agents had not yet been incorporated into Ecuador’s national essential medicines list at the time of data collection. The results should be interpreted as hypothesis-generating and warrant confirmation in prospective, multicentre studies.

In conclusion, “Los Ceibos” chronic HF registry demonstrated high prescription rates of ACEis/ARBs and BB; however, this was not observed with ARNIs. The use of triple foundational therapy, specifically the combination of ARNI + BB + MRA, was associated with improved prognosis in patients with HFrEF over a four-year follow-up period.
